# Cancer-Associated Fibroblast Heterogeneity and Its Influence on the Extracellular Matrix and the Tumor Microenvironment

**DOI:** 10.3390/ijms241713482

**Published:** 2023-08-30

**Authors:** Karl Knipper, Su Ir Lyu, Alexander Quaas, Christiane J. Bruns, Thomas Schmidt

**Affiliations:** 1 Department of General, Visceral and Cancer Surgery, University Hospital of Cologne, Faculty of Medicine, University of Cologne, 50937 Cologne, Germany; karl.knipper@uk-koeln.de (K.K.); christiane.bruns@uk-koeln.de (C.J.B.); 2 Institute of Pathology, University Hospital of Cologne, Faculty of Medicine, University of Cologne, 50937 Cologne, Germany; su.lyu@uk-koeln.de (S.I.L.); alexander.quaas@uk-koeln.de (A.Q.)

**Keywords:** fibroblasts, myofibroblasts, iCAFs, eCAFs, pericytes, extracellular matrix, targeted therapy

## Abstract

The tumor microenvironment comprises multiple cell types, like cancer cells, endothelial cells, fibroblasts, and immune cells. In recent years, there have been massive research efforts focusing not only on cancer cells, but also on other cell types of the tumor microenvironment, thereby aiming to expand and determine novel treatment options. Fibroblasts represent a heterogenous cell family consisting of numerous subtypes, which can alter immune cell fractions, facilitate or inhibit tumor growth, build pre-metastatic niches, or stabilize vessels. These effects can be achieved through cell–cell interactions, which form the extracellular matrix, or via the secretion of cytokines or chemokines. The pro- or antitumorigenic fibroblast phenotypes show variability not only among different cancer entities, but also among intraindividual sites, including primary tumors or metastatic lesions. Commonly prescribed for arterial hypertension, the inhibitors of the renin–angiotensin system have recently been described as having an inhibitory effect on fibroblasts. This inhibition leads to modified immune cell fractions and increased tissue stiffness, thereby contributing to overcoming therapy resistance and ultimately inhibiting tumor growth. However, it is important to note that the inhibition of fibroblasts can also have the opposite effect, potentially resulting in increased tumor growth. We aim to summarize the latest state of research regarding fibroblast heterogeneity and its intricate impact on the tumor microenvironment and extracellular matrix. Specifically, we focus on highlighting recent advancements in the comprehension of intraindividual heterogeneity and therapy options within this context.

## 1. Introduction

Despite massive research efforts, cancer remains a global healthcare challenge. Projections anticipate that, in the year 2022 alone, in the United States of America, around two million patients were diagnosed with cancer, and 609,360 patients suffered cancer-related deaths [1]. Published in 2000, Hanahan et al. introduced the hallmarks of cancer, which represent the main acquired capabilities for the progression of cancer known at that time [2]. Over twenty years later, due to extensive research, this list has been enlarged to currently 14 main capabilities [3]. While initially, the research was centered around tumor cells, the focus was broadened to the tumor microenvironment (TME), including all cell types present in the tumor. It not only became obvious that the tumor microenvironment plays a crucial role in tumor progression, but also that a better understanding of its function could lead to novel therapeutic targets. The tumor microenvironment consists of fibroblasts, endothelial cells, and immune cells, such as granulocytes, B and T lymphocytes, macrophages, mast cells, natural killer cells, dendritic cells, and the extracellular matrix [4,5]. These cells could be resident cells, or they could infiltrate the tumor tissue due to the chemoattractants that are secreted by the tumor cells or other cellular components of the TME [4]. The microenvironment cells can act either pro- or antitumorigenically. For instance, macrophages showcase a heterogenous behavior in their function in the context of the tumor microenvironment. Resident macrophages in the liver show cytotoxic abilities, thus preventing metastases [6]. On the contrary, protumorigenic signals of the tumor cells could even be aggravated by macrophages via the secretion of additional cytokines [7].

A comprehensive analysis of the tumor microenvironment in patients with breast ductal carcinoma in situ (DCIS) has shed light on crucial insights. Specifically, comparing patients who experienced relapse to those who did not, significant disparities in cellular composition and spatial distribution were identified. Moreover, the cellular composition differs when compared to normal tissue, ductal carcinoma in situ, and invasive breast cancer [8].

Fibroblasts represent another heterogeneous cell family involved in the tumor microenvironment. They play a crucial role in physiological processes like wound healing, but they are also involved in some pathological events [9,10,11]. A broad variety of fibroblast markers is known: for instance, α-smooth muscle actin (αSMA), fibroblast activation protein (FAP), periostin, or platelet-derived growth factor receptor-β (PDGFR-β). These can be used to distinguish between different fibroblast subtypes. As a juxtaposition to the earlier ages of cancer treatment, which inhibit all fast-proliferating cells in the tumor, targeted treatment strategies are emerging and could potentially not only reduce the number of adverse events, but also—at the same time—provide more potent therapy options [12]. Distinct fibroblast subtypes represent possible targets for these novel treatment options. Exemplary monoclonal antibodies against FAP have shown an inhibition of tumor progression in mouse models of head, neck, lung, and pancreatic cancer [13].

In addition to the abovementioned cell types, the tumor microenvironment consists of, in comparison with the normal tissue-altered extracellular matrix, a protein composition that is produced by all cell types present in the tumor. This matrix typically contains a collagen network [14]. The extent of the latter differs between the different solid tumors and influences the ability of macromolecules to penetrate the tumor tissue, which leads to mechanical anti-tumor drug resistance in some solid tumors [15].

In this review, we describe the cell family of fibroblasts and their role in tumor progression, as well as their influence on building extracellular matrix in tumors. Additionally, we summarize recent advancements in the development of new treatment options that target fibroblasts and extracellular matrix proteins.

## 2. Cancer-Associated Fibroblasts

Cancer-associated fibroblasts (CAFs) can be classified according to their marker expression. αSMA, for instance, is a typical fibroblast marker, which occurs not only in cancer-associated fibroblasts, but also in those involved in physiological mechanisms such as wound healing [16,17]. However, taking into consideration that not all fibroblasts demonstrate a singular marker profile, various markers can be used to label distinctive fibroblast subgroups. The combination of several fibroblast markers enables further subdivision of the fibroblast population, which could, among other things, potentially help to predict the tumor disease outcome. For instance, in pancreatic ductal adenocarcinomas (PDAC), the specific combination of expression levels, such as Periostin^high^SMA^high^ or Periostin^high^SMA^low^PDGFR^low^FAP^high^, showed a correlation with worsened patient survival [18]. In colorectal cancer, a specific CAF gene signature, compared with the fibroblasts of normal tissue, correlated with worsened patient survival, therefore highlighting fibroblasts as a possible biomarker for worse survival rates [19].

Additionally to the measurement of marker expression levels, the differentiation of the fibroblast composition in the tumor microenvironment could be analyzed through different tumor morphologic appearances. To illustrate this phenomenon, fibroblasts from head and neck squamous cell cancers, both those with and without a demarcation zone between epithelial cells and the extracellular matrix, were examined. Here, fibroblasts within tumors exhibiting a demarcation zone demonstrated a significant reduction in their ability to stimulate tumor cell invasion, coupled with a simultaneous significant increase in the expression of distinct collagen types (e.g., COL26A1) [20]. Confirming the described abilities of distinct fibroblast subgroups, high SMA, PDGFR-β, or collagen-I could be correlated with venous invasion, whereas D2-40 did not show a significant correlation [21]. Furthermore, elevated levels of Lipoma preferred partner (LPP), expressed by CAFs, were identified as an independent factor for worse survival in gastric cancer. Additionally, heightened levels of its expression are associated with unfavorable responses to established chemo- or immunotherapy protocols. Using the GDSC dataset, the authors could estimate the therapeutic response of 28 chemical compounds for patients with high LPP expression [22].

*CAFs Demonstrate a Heterogenous Cell Family*

Single-cell characterization, such as single-cell RNA sequencing, has notably facilitated the detection of various cell types, including fibroblast subtypes [23]. Li et al. performed single-cell RNA sequencing along with immunohistochemical multi-stainings in tissue samples obtained from both gastric cancer and healthy mucosa. The synergic combination of the two methods has provided valuable spatial information, shedding light on the dimensional positioning of fibroblasts in relation to other cell types. Moreover, the study revealed significant intratumoral fibroblast variability.

**Figure 1:**
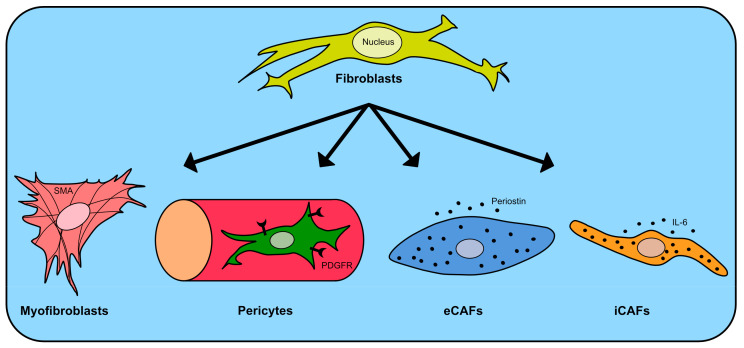
Overview of the four main fibroblast subgroups—myofibroblasts, pericytes, extracellular matrix CAFs (eCAF), and inflammatory CAFs (iCAF)—depicting their main markers. IL-6: Interleukin 6, PDGFRβ: Platelet-derived growth factor receptor-β, and SMA: α-smooth muscle actin.

Four main fibroblast clusters within cancerous tissue, each possessing distinct characteristics, have been described (Figure 1 and Table 1): myofibroblasts, pericytes, extracellular matrix CAFs (eCAF), and inflammatory CAFs (iCAF). Notably, upon comparing these fibroblast subgroups within the tumor microenvironment to their counterparts in the normal mucosa, a discernible observation emerged: fibroblasts within the tumor showcased elevated protumor characteristics. This differentiation was established by analyzing the varying expression levels of genes pivotal in promoting proliferation or angiogenesis. iCAFs were shown to be enriched in the tumor gland and lymphoid nodule-like areas and displayed interaction properties with the T lymphocytes. On the contrary, eCAFs were prevalent in the distal stroma area and influenced the protumor M2 macrophages [24].

*Extensive Crosstalk between Fibroblast and Tumor Microenvironment Alters Patients’ Outcome*

In recent years, further data regarding the crosstalk between fibroblasts and other cell types of the TME has emerged. The deletion of collagen I, which is mainly produced by fibroblasts in PDAC, aggravates the inhibition of B and T lymphocytes, therefore supporting the tumor progression [25].

Moreover, the depletion of myofibroblasts in pancreatic cancer leads to elevated levels of regulatory T lymphocytes while simultaneously resulting in a reduction of effector T lymphocytes. This imbalance ultimately leads to reduced patient survival rates [26]. In bladder cancer, CAFs can be activated by interferon, a cytokine secreted by tumor cells. This subgroup of fibroblasts is characterized by the urea transporter SLC14A1. These activated CAFs confer tumor cell stemness via Wnt-5a secretion. Patients exhibiting a higher proportion of SLC14A1-positive CAFs tend to experience unfavorable outcomes and show diminished responses to therapy [27].

In esophageal squamous cell cancer, the levels of Annexin A1 (ANXA1), which is secreted by epithelial cells, decrease as the tumor advances. Under physiological conditions, this protein plays an important role in preserving fibroblast balance. However, suppression of ANXA1 triggers the transformation of normal fibroblasts into CAFs, a change that is magnified by the malignant cells’ secretion of TGF-beta [28]. CAFs can support cancer cell stemness, epithelial–mesenchymal transition (EMT), metastasis, and therapy resistance via direct transfers of exosomes into colorectal cancer cells. This results in the activation of the Wnt pathway [29]. Additionally, cancer-associated fibroblasts (CAFs) play an important role in helping tumor cells to adapt to a glutamine-deprived microenvironment. CAFs are capable of autophagy, particularly ribophagy, which relies on the nuclear fragile X mental retardation-interacting protein 1 (NUFIP1) in pancreatic cancer cells. As a result, CAFs secrete nucleosides that are subsequently absorbed by tumor cells. This enhances the glucose utilization of cancer cells both in vitro and in vivo. [30]. Another subpopulation of CAFs is defined as CD10^+^GPR77^+^ fibroblasts, which are activated by NF-κB [31].

Both markers are less commonly utilized. CD10, a membrane metalloendopeptidase, is found on CD10^+^ fibroblasts, which have been observed to promote specific tumor cells [32]. On the other hand, G-protein-coupled receptor 77 serves as a regulator of immune function [33]. CD10^+^GPR77^+^ fibroblasts promote cancer stem cells and, therefore, tumor chemoresistance by secreting interleukin 6 and 8. Inhibition of CD10^+^GPR77^+^ fibroblasts with a neutralizing anti-GPR77 antibody restores the chemosensitivity [31].

*Myofibroblasts (myCAFs)*

Myofibroblasts represent a widely known subgroup of fibroblasts [34], characterized by the expression of SMA [17]. The differentiated myCAFs may have a multilocular origin—bone marrow-derived or deriving of resident cells like fibroblasts or hepatic stellate cells [35,36]. Hepatic stellate cells (HSC) show a dual polarization. Cytokine-producing HSCs can inhibit hepatocellular carcinoma development. In contrast, myCAFs, which are evolved from HSCs, promote tumor progression. The HSC subpopulations shift towards myCAFs during the progression of liver fibrosis and cirrhosis [37]. Similar findings regarding the change of subpopulations during tumor progression but also under immune checkpoint therapy or mechanical force could be detected in breast cancer, skin cancer, and pancreatic cancer [38,39]. Interestingly, recent studies revealed a myCAF subset, which origins from the macrophage lineage. This macrophage–myofibroblast-transformation can be observed in non-small-cell lung carcinoma and is associated with worse patients’ survival. Further investigations identified Smad3 as a main factor of this transition [40]. The activation of this specific subgroup is triggered by the autocrine secretion of the transforming growth factor β (TGF-β) [36,41]. Further cofactors have been recently identified. RAS-responsive element binding protein 1 (RREB1) functions as a crucial partner of TGF-β, which results in the activation of Smad and, consecutively, Zinc finger protein SNAI1. This activates the epithelial-to-mesenchymal transition, myofibroblasts, intratumoral fibrosis, and tumor progression [42]. The main roles of myCAFs are traction regulation and altering collagen rearrangement [43,44]. myCAFs are involved in physiological, but also in pathological mechanisms, such as cancer progression or fibrosis [45,46]. Myofibroblasts could be shown to play an unfavorable role in the cancer progression of patients with ductal breast cancer. Additionally, elevated levels of basic fibroblast growth factor, Ki-67, and vascular endothelial growth factor (VEGF) could be detected in tissue samples with higher myCAF infiltration. This could implicate a promoting role in angiogenesis and cell proliferation of myCAFs in the tumor microenvironment [47]. Further investigations could reveal that leucine-rich-repeat-containing protein 15-positive CAFs (LRRC15) represent the main part of CAFs in pancreatic cancer. Depletion of LRRC15 leads to a more physiological fibroblast population in cancer in vivo. Consecutively, CD8^+^ T lymphocytes are less suppressed, and immune checkpoint inhibitors demonstrate higher effectiveness [48]. Confirming this immune-suppressing ability of myCAFs, tryptophan 2,3-dioxygenase-positive myCAFs (TDO2) have been identified in oral squamous cell carcinoma. This subset of myCAFs induces the transition of CD4^+^ T lymphocytes into regulatory T lymphocytes and causes dysfunction of CD8^+^ T lymphocytes [49]. C-X-C motif ligand 3 (CXCL3), which is mainly secreted by macrophages, induces the fibroblast to myofibroblast transformation. This induces the expression of migration- and invasion-related genes. Furthermore, these myCAFs can induce metastasis in pancreatic cancer [50]. A higher amount of myCAFs is associated with larger tumor sizes and poor survival in cholangiocarcinoma [51]. This tumor promotion is associated with the expression of hyaluronan synthase 2 of myCAFs in cholangiocarcinomas [52]. The appearance of myCAFs is associated with lymph node metastases in colorectal cancer. Moreover, the myCAF density is positively correlated with the size of the metastasis [53]. While myCAFs can promote tumor progression via the secretion of hyaluronan, they can also secrete collagen I, which results in mechanical tumor growth suppression [54]. It is evident that myofibroblasts maintain complex interactions with cancer cells. Here, myCAFs in cholangiocarcinoma have been shown to secrete matricellular proteins like Periostin, growth factors like hepatocyte growth factor, and therefore activate pathways like PI3K/Akt or Hedgehog (Hh)-GLI [55], which leads to tumor progression and therapy resistance [56].

*Pericytes*

Pericytes are a further subgroup of fibroblasts, showcasing an immense variability. The morphology of these cells, located on vessels like capillaries, arterioles, and venules, differs drastically depending on the vessel type [57]. The physiological functions of pericytes include maintaining vasculature stability, blood flow control, and vasculature permeability [58]. Pericytes are also involved in various pathologic mechanisms and are suspected to promote tumor angiogenesis [59,60,61]. In serous ovarian cancer, having a high number of pericytes is associated with poorer survival outcomes for patients. Additionally, when pericytes and ovarian cancer cells were co-injected in xenograft models, there was a significant increase in tumor growth and metastasis progression [62]. Platelet-derived growth factor-B (PDGF-BB) induces the so-called pericyte-fibroblast transition, which marks the detachment of pericytes of the vasculature and differentiation to stromal fibroblasts. Knockdown or pharmaceutical inhibition of PDGF-BB leads to increased pericyte coverage of vasculature and decreased stromal fibroblasts in vitro and in vivo. This leads to tumor inhibition [60]. Interestingly, similar effects can be seen in prostate and lung cancer models following radiotherapy. Pericyte coverage of the vasculature is significantly impaired after radiation, which leads to decreased hypoxia and enhanced perfusion [63]. Additionally, the pericyte-fibroblast transition results in microvessel leakage and inflammatory reaction. Blocking the transition, mainly regulated by PDGF-BB, with imatinib decreases scarring, as well as inflammatory reactions [64].

Pericytes interact to a great extent with other cell types of the tumor microenvironment. To maintain vasculature stability, a close interaction between pericytes and endothelial cells is needed. Abolishing this interaction leads to disruption and deregulation of vessels. The upregulation of prostaglandin E2 triggers downregulation of adherents and gap junctions [65,66]. Cell invasion—for example, of immune cells or cancer cells—requires a distinct leakiness of vessels, and pericytes play a crucial role in vessel stability. Cancer cells can induce a pericyte transition to SMA-positive pericytes by exosomes. Consistent with this assumption, these altered pericytes are found to be significantly enriched, particularly in pancreatic adenocarcinoma. Further investigations revealed that SMA-positive pericytes are correlated to an increased leakiness of vasculature. Additionally, the immunoregulatory phenotype of these pericytes was altered [67].

Pericytes play a significant role as contributors to the premetastatic niche by including proliferation, migration, and synthesis of the extracellular matrix [68]. In addition to communication between cancer cells and pericytes through chemokines or cytokines, contact-dependent interactions also exist between pericytes and cancer cells. Cdc42-dependent contacts lead to recruiting of preexisting vessels and, therefore, to tumor progression in glioblastoma. Furthermore, inhibition of Cdc42 was followed by pericytes initiating an immune response, which suppressed tumor growth in vitro and in vivo [69]. This suggests that pericytes make out a heterogenous subpopulation, which can—depending on the cell–cell interaction—have pro- or antitumorigenic characteristics. As an expression of this heterogenous subpopulation, pericytes were under suspicion of promoting therapy resistance against antiangiogenic therapy. To further investigate this theory, lung cancer-bearing pericyte-deficient mice were treated with an anti-VEGF-A antibody. Treatment success was measured with tumor growth and vascular density. However, compared to control mice, no further treatment success was observed [70]. In addition to neoangiogenesis, aggressive cancer cells can establish a vasculature-like formation, known as vascular mimicry. Here, pericytes induce sprouting and provide stabilization of the formed vasculature-like structures. Pharmaceutical blocking of PDGF-B inhibits vascular mimicry and tumor growth [71].

*Inflammatory CAFs (iCAFs)*

Inflammatory reactions are regulated through various pathways, including cytokines and growth factors. [72]. These can be secreted by different cell types, amongst others fibroblasts [73]. Fibroblasts can influence immune reactions directly by secreting cytokines or, alternatively, by altering the extracellular matrix [73,74]. These changes in the tumor microenvironment can be initiated by tumor cells. Tumor cell-derived exosomes initiate the differentiation and proliferation of CAFs in head and neck squamous cell carcinoma. By secreting cytokines, these fibroblasts contribute to a reduction in T-cell infiltration and an increase in the presence of protumoral macrophages within the tumor microenvironment. [75]. Mammary cancer neoplastic cells can induce fibroblast reprogramming by secreting Osteopontin, a proinflammatory mediator. Consequently, fibroblasts alter the tumor microenvironment and extracellular matrix by augmenting collagen secretion and releasing proinflammatory mediators. Inhibiting Osteopontin has led to tumor growth inhibition in mice models [76]. Accordingly, Nuclear factor kappa B (NF-κB) is also capable of inducing fibroblast differentiation into iCAFs in skin cancer. These iCAFs are defined as proinflammatory gene signature, which leads to macrophage recruitment, tumor progression, and neoangiogenesis [77]. iCAFs are not solely activated by cancer cell-dependent cytokine secretion. Additionally, due to hypoxic conditions, CAFs can be reprogrammed to iCAFs by the mediator HIF1α and, therefore, accelerate tumor growth [78]. Öhlund et al. showed that CAFs are responsible for high IL-6 levels in conditioned media of co-cultures of fibroblasts and cancer cells in vitro [79]. Interleukin 6 is known to suppress hepatic ketogenesis in tumor-bearing mice, leading to higher glucocorticoid secretion, immunosuppression, and failure of the immunotherapy [80]. Flow cytometry showed that two different fibroblast subtypes exist in PDAC: myofibroblasts and IL-6 expressing fibroblasts, inflammatory CAFs. These iCAFs represent the main source of IL-6 in organoids of PDAC. In vivo immunohistochemistry demonstrated that myCAFs are mainly located next to cancer cells, whereas iCAFs are more distantly from neoplastic cells [79]. Accordingly, fibroblasts, which express cytokines and chemokines like IL-6, CXCL12, or insulin-growth-factor 1 (IGF1), could be detected in bladder urothelial carcinoma. The abundance of specific cell types in the tumor microenvironment was correlated with patients’ survival. Interestingly, a higher infiltration of iCAFs was correlated with poor survival [81]. iCAFs are associated with intrahepatic cholangiocarcinoma growth. This effect seems to be established by the secretion of hepatocyte growth factor by myCAFs, which leads to protumorigenic activation of MET [52]. Furthermore, higher amounts of iCAFs were associated with the stronger infiltration of macrophages and T lymphocytes, as well as with the incidence of mutations of genes like TP53 [82]. In line with the previously mentioned results, iCAFs are associated with worse survival in patients with rectal cancer. Nicolas et al. showed in a mice model of rectal carcinoma that tumor cells are causing a fibroblast polarization towards iCAFs by IL-1. These iCAFs are contributing to tumor therapy resistance. Thus, inhibition of IL-1 impairs tumor response. These results could be verified by detecting lower serum levels of the antagonist of IL-1 in patients with non-complete responses, resulting in worse patient survival [83].

*Fibroblast Subpopulations Can Overlap*

CD105 distinguishes two different fibroblast lineages in pancreatic ductal adenocarcinoma (PDAC). CD105^+^ and CD105^−^ fibroblasts express markers of both subpopulations, myofibroblasts and inflammatory fibroblasts. CD105^−^ fibroblasts show tumor-suppressing characteristics in PDAC by inducing adaptive immunity in contrast to CD105^+^ fibroblasts [84].

*Extracellular Matrix CAFs (eCAFs)*

Extracellular matrix cancer-associated fibroblasts sum up another recently discovered major fibroblast subpopulation characterized by the expression of Periostin [85,86]. Initially described, Malanchi et al. identified a Periostin-expressing, tumor-derived fibroblast subpopulation in the lung metastasis sites, which did not alter tumor growth or cancer cell survival. However, these fibroblasts were crucial for the maintenance of cancer stem cells and for building the pre-metastatic niche in secondary target organs [85]. Other studies identified a subpopulation of fibroblasts, which is characterized by the expression of genes related to extracellular matrix remodeling [87]. eCAFs could also be detected in gastric cancer. Here, eCAFs are significantly associated with worse patient survival. In contrast to iCAFs, eCAFs induce migration of protumorigenic-M2 macrophages via the secretion of Periostin. Further investigations could prove a spatial relationship between eCAFs, which mainly exist in the distant stroma area, and M2 macrophages. Additional gene analysis revealed an upregulation of genes related to the tumor invasion, like matrix metalloproteinase 14 (MMP14) or lysyl-oxidase like 2 (LOXL2) [24]. Notably, cell–cell interactions are particularly discernible between myofibroblasts and eCAFs. [86]. Investigation of the abundance of eCAFs and therapy response in patients with gastric cancer revealed the prognostic value of eCAFs for poor response to anti-PD1 treatment. The activation of Akt signaling pathways in macrophages, induced by the secretion of Periostin from eCAFs, could potentially serve as a causal factor leading to alterations in the tumor microenvironment [88].

*Metastasis-Associated Fibroblasts*

The tumor microenvironment differs drastically between various cancer entities. However, these differences can be observed not only between separate entities but also in the same cancer entity and, furthermore, even in the same patient depending on different tumor sites, as presented in metastasized patients [89,90,91]. In the following paragraph, we further characterize the state of research regarding metastasis-associated fibroblasts (MAFs).

*MAFs and CAFs Express Different Genes*

MAFs can exhibit distinct abilities and characteristics when compared to CAFs. Metastasis-associated fibroblasts showed a higher proportion of myofibroblasts in colorectal liver metastasis in comparison to the primary tumor. Gene expression analyses between primary CAFs and MAFs were performed and exposed to different expression levels in these two groups. MAFs presented an enrichment of genes of angiogenesis and myogenesis. Quantifying the stiffness of human tissue revealed that liver metastases are significantly stiffer than the colorectal primary tumor. Furthermore, stiffness is positively correlated with angiogenesis [91]. Fibroblasts provide substantially to the extracellular matrix and, therefore, influence the tissue stiffness [92]. Indeed, MAFs contracted the ECM in in vitro gel contraction models more than CAFs [91]. Furthermore, cell–cell interactions in metastasis differ compared to the primary tumors. An increase in cell–cell interaction between myofibroblasts and eCAFs could be detected in metastasis of oral squamous cell carcinoma. eCAFs induce a significant upregulation of Wnt and Notch pathways in cancer cells in metastatic lesions. Furthermore, eCAFs show an increased response to cytokines and extracellular matrix organization in metastasis [86].

*MAFs Promote Cancer Cell Migration and Proliferation*

One hallmark of developing metastasis is the ability of cancer cells to migrate into other tissues by extravasation [93]. The blood–brain barrier is built by astrocytes, endothelial cells, and pericytes. Here, pericytes play a crucial role in inhibiting the development of brain metastasis. Pericytes provide not only a mechanical barrier by forming tight junctions but also inhibit the ability of lung cancer cells to proliferate [94]. Contrarily, pericytes can also facilitate brain metastasis by promoting adhesion and proliferation, as observed in breast cancer. Pericytes-conditioned medium leads to increased proliferation of the breast cancer cells. Further investigations revealed that insulin-growth-factor 2 (IGF2), which is secreted by pericytes, is one of the factors responsible for the increase of proliferation of breast cancer cells. Silencing and inhibition of IGF2 led to decreased proliferation. Furthermore, cancer cells seeded in a pericytes-conditioned medium showed higher phosphorylation of focal adhesion kinase and Src, which in turn regulate proteins for adhesions [95]. Apparently, this effect is dependent on the tumor entity. Similar experiments with melanoma cells showed less influence of pericytes on these cancer cells [96]. Co-cultures of MAFs and metastatic pancreatic cancer cells have shown enhanced angiogenesis due to the secretion of CXCL8 and CCL2 by fibroblasts [97].

*MAFs Alter the Immune Cell Composition in Tumors*

Fibroblasts play a significant role in metastasis by modulating the composition of the tumor microenvironment. Fibroblast growth factor 2 (FGF2), secreted by nasopharyngeal cancer cells, promotes pericyte proliferation. Consecutively, pericytes secrete C-X-C motif ligand 14. This ends in macrophage polarization towards M2-like macrophages, known to promote tumor progression. These macrophages facilitate tumor migration. Thus, inhibiting FGF2 leads to a decrease in pulmonary metastasis [98].

Taken together, fibroblasts interact with cancer cells greatly during the initiation and progression of metastasis. Based on the subgroup of fibroblasts and the tumor entity, fibroblasts can act pro- or antimetastatic.

## 3. Extracellular Matrix (ECM)

The extracellular matrix consists of various proteins, which are mainly secreted by fibroblasts [99].

*Tumor Stiffness Differs in Gene Expression and Pathway Activation*

Among other observations, the fibroblast composition also influences the tumor stiffness [100]. This alters known hallmarks of cancer, like neoangiogenesis, cell invasion, or cell proliferation [14].

Higher stiffness could be caused by increased collagen bundling or the formation of crosslinks. An increase in collagen bundles leads to higher cancer cell invasion in organoids. In contrast, more crosslinks are leading to a decrease in the collective invasion of cancer cells [101]. Tumor stiffness could also be modified by medicaments without altering the collagen composition. Anti-VEGF therapy leads to an increase in hyaluronic acid and sulfated glycosaminoglycans in mouse models of colorectal liver metastases. Additional enzymatic depletion of hyaluronic acid could provide prolonged survival under anti-VEGF therapy [102]. The stiffness of the extracellular matrix is controlled by numerous pathways and is highly complex [103,104]. In mesenchymal stem cells of the bone marrow and primary chondrocytes, higher ECM stiffness showed an increased activation of the Wnt/β-catenin pathway, amplified by a positive feedback loop [105]. Upregulation of the Wnt pathway plays a crucial role in tumor progression in several cancer entities [106]. It could be shown that distinct ribonucleic acids (RNA) are enriched in APC protein-dependent (adenomatous polyposis coli) matter in specific cell protrusions. These protrusions showed a significantly higher stiffness, which supported an increase in cell migration [107].

*Collagens Are a Main Part of the ECM*

A big protein family of collagens is coming into the focus of cancer research as a biomarker, as well as a potential treatment target [108]. The ECM with collagen as the main player is greatly involved in cell migration and metastatic progression [109,110]. In vitro experiments could detect proteins of the ECM like collagen I, collagen III, fibronectin, and laminin 421 as activators of cell migration of breast and prostate cancer cells [111]. Collagen I accumulation showed no differences in primary tumor growth in estrogen receptor alpha-positive mammary tumors. However, significantly more circulating cancer cells and a higher metastatic burden could be detected in a mice model [109]. However, the role of collagen I has not been completely understood yet. Collagen I, which is mainly produced by myCAFs in colorectal and pancreatic liver metastasis, suppresses tumor progression via mechanical restraining cancer cell spreading [54].

Fibroblasts do not solemnly contribute to collagen synthesis for ECM. Adipocytes secrete collagen VI in breast cancer. This adipocyte-derived collagen VI influences tumor formation and primary tumor progression [112]. Distinct collagen expression in the ECM can lead to therapy resistance. Collagen VI supports cisplatin resistance in vitro in ovarian cancer cells. Moreover, poorly differentiated ovarian tumors showed significantly higher collagen VI expression levels [113]. The production of collagen by fibroblasts can be inhibited by losartan, a widely used angiotensin II receptor antagonist. By inhibition of collagen synthesis of fibroblasts, losartan improved the treatment response to injected antitumorigenic herpes simplex viruses and doxorubicin [114].

*Fibroblasts Subgroups Secrete Different Proteins of the ECM*

Fibroblasts secrete extracellular matrix proteins based on their polarization and interactions with cancer cells. Pericytes secrete high amounts of collagen type IV and fibronectin [96]. iCAFs have a higher gene expression signature for extracellular matrix organization compared to myCAFs [81]. Hyaluronan and proteoglycan link protein 1 (HAPLN1) is shown to be upregulated in CAFs in gastric cancer. Higher levels of HAPLN1 are correlated with worse survival. Pathomechanically, HAPLN1 facilitates tumor cell migration and invasion [115].

*Environmental Conditions Alter ECM*

Moreover, other factors can contribute to variations in the secretion pattern of fibroblasts, consequently affecting the composition and integrity of the extracellular matrix. After irradiation, the ECM is enriched with distinct glycoproteins, collagens, proteoglycans, and growth factors known for their protumorigenic abilities [83]. Further, hypoxic conditions, as often present in progredient tumors, can also lead to an altered ECM. This could either facilitate collagen synthesis or degradation. Fibroblasts are activated by transforming growth factor-β [116,117].

*ECM Synthesis and Degradation Are Existing Simultaneously in Cancer*

The production and degradation of extracellular matrix proteins occur concurrently. The degradation process is mainly mediated by matrix metalloproteinases (MMPs). These enzymes are primarily secreted by fibroblasts; however, they can also be expressed by macrophages or neutrophils [118]. However, the secretion of MMPs and maintaining the equilibrium of the ECM requires highly complex regulating mechanisms. MMP-2 and MMP-9 regulate the ability of cancer cell invasion in human head and neck cancer. This seems to be induced by CXCL12. Noteworthy, activation of these proteinases needs direct cell contact between tumor cells and fibroblasts [119]. This indicates that cell-matrix interactions and cell–cell interactions are required for the regulation of ECM in cancer. CAF-secreted MMP-2 induced a solving of cohesions of keratocytes, which supports epithelial invasion in oral squamous cell carcinoma [120]. MMP-1 shows higher expression levels in triple-negative breast cancer. A knockdown of MMP-1 results in decreased cancer cell proliferation and migration [121]. An upregulation of MMP-1 in CAFs, which are co-cultured with breast cancer cells, revealed a loss of efficacy of docetaxel, an anti-tumor treatment drug. However, this loss could be reversed by inhibition of MMP-1. Interestingly, a higher MMP-1 level correlated with higher levels of collagen IV [122]. MMPs can also directly support tumor growth. MMP-13 induces VEGF-A secretion of fibroblasts and endothelial cells. This leads to increased angiogenesis in vitro and in vivo. Additionally, MMP-13 expression correlated with blood vessel density in human head and neck squamous cell carcinoma samples [123]. This reveals that MMPs can support tumor progression by facilitating cell invasion and inducing angiogenesis.

## 4. Therapy

As discussed above, fibroblasts carry out diverse roles depending on cancer entities and fibroblast subgroups. Several subgroups correlated with worse survival were identified (Table 2). This protumorigenic effect of fibroblasts was associated with the activation of cancer cell migration, proliferation, and angiogenesis, causing therapy resistance. Therefore, developing targeted treatment options against fibroblasts has gained importance.

**Figure 2:**
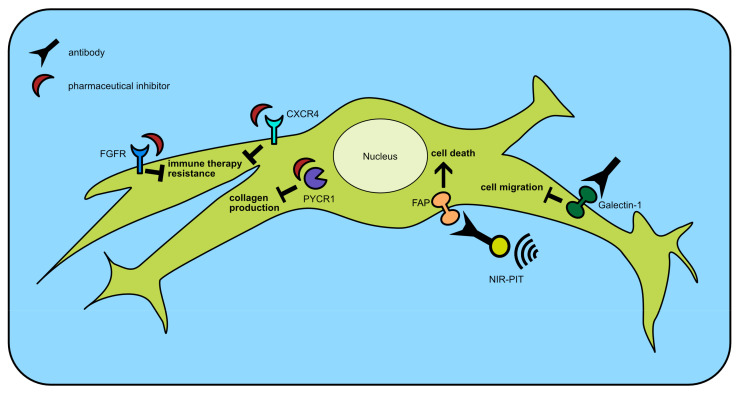
Recently developed therapy options to inhibit fibroblasts or ECM. CXCR4: C-X-C motif receptor 4, FAP: Fibroblast activation protein, FGFR: Fibroblast growth factor receptor, PYCR1: Pyrroline-5-carboxylate reductase 1, and NIR-PIT: Near-infrared photoimmunotherapy.

Cancer cells are highly unstable due to frequent gene mutations and differ between patients a lot. Therefore, non-mutated cells like fibroblasts could be an ideal treatment target. Figure 2 gives an overview of the recently developed and below-described therapy options.

*Inhibition of Specific Fibroblast Markers Suppresses Tumor Progression*

In oral squamous cell carcinoma, the infiltration of myCAFs has been found to positively correlate with Galectin-1 expression. Galectin-1, a member of the β-galactoside-binding lectin family, is located on the cell surface and is able to recognize a wide range of molecules. Further, in vitro investigations presented that Galectin-1 is crucial for the activation of myCAFs. An inhibition of Galectin-1 results in decreased tumor-cell migration, invasion, and tumor progression. Galectin-1 possibly regulates tumor progression through the monocyte chemotactic protein-1. Targeting this protein with an antibody in vitro has led to less cell invasion [124]. Major signal pathways are involved in fibroblast and cancer cell communication. Targeting parts of these pathways could lead to tumor suppression. CD248, which is expressed by tumor-associated stromal cells, is coexpressed by two angiogenic factors (OPN and SERPINE1) in lung cancer models. Higher levels of these factors could be associated with worse survival of lung cancer patients. Mice lacking CD248 exhibit reduced tumor volume and vessel density. It is worth highlighting that these CD248-deficient mice also display developmental abnormalities. This suggests the significance of CD248 and that its inhibition might lead to adverse events and, therefore, should be handled with great consideration. Mechanistically, increased stimulation of the Wnt/β-catenin-pathway leads to higher levels of OPN and SERPINE1. The effect of the genetic deletion can be mimicked by β-catenin-inhibitor [125].

*Novel Targeted Therapies Such as Near-Infrared Photoimmunotherapy and Chimeric Antigen Receptor T Cells against Fibroblasts Could Improve Patients’ Survival*

Innovative treatment strategies, including chimeric antigen receptor T cells, were also rolled out to target stromal cells. Here, the specific deletion of FAP-positive fibroblasts led to tumor inhibition in subcutaneously mice models of lymphoma, mesothelioma, and breast, colon, and lung adenocarcinoma [126]. Near-infrared photoimmunotherapy (NIR-PIT) is another novel therapy regime. Here, specific targeted antibodies, which are conjugated with a photosensitizer, are administered. The cytotoxic effect is developed by applying near-infrared light. Numerous in vitro and in vivo studies have shown promise in investigating the impact of targeting CAFs using NIR-PIT [127,128,129,130]. NIR-PIT proved to overcome therapy resistance in esophageal cancer. The combination of fluorouracil and FAP-targeted NIR-PIT resulted in a 70.9% tumor reduction compared to only 13.3% by fluorouracil alone [129]. Additionally, the combination of EGFR- and FAP-targeted NIR-PIT achieves suppression of tumor growth in vivo [128]. These studies are limited due to their reliance on immune-deficient mice models, which hinders the assessment of immune responses following therapy administration. Furthermore, long-term outcomes are absent in the mentioned studies. Subsequent investigations should overcome these limitations by altering the study design. A key advantage of this innovative treatment lies in its versatility to target a wide array of proteins. Thus, once established, it could be easily adapted for diverse treatment targets, including other cells of the tumor microenvironment.

*Inhibition of ECM Production in CAFs*

It is known that ECM can greatly alter tumor progression. Therefore, targeting certain proportions of ECM could lead to a novel targeted therapy option. Kay et al. have shown that proline must be synthesized by CAFs for collagen synthesis. Pyrroline-5-carboxylate reductase 1 (PYCR1) is one of the key enzymes in the production of proline. Inhibition of PYCR1 resulted in a significant reduction of collagen production, tumor progression, and metastatic burden in a murine breast cancer model [131].

*Targeting CAFs Helps to Overcome the Chemoresistance*

Fibroblasts play a significant role in the development of treatment resistance, with one mechanism involving the regulation of circular RNA (circRNA) of the FERM, RhoGEF, and pleckstrin domain-containing protein 1 (FARP1). circFARP1 alters gemcitabine resistance in pancreatic cancer via the leukemia inhibitory factor. High expression levels of circFARP1 could be correlated with worse patients’ survival and gemcitabine resistance [132]. Using a DNA vaccine against FAP leads to a CD8^+^ T cell-mediated elimination of fibroblasts. This revealed suppression of primary tumor and metastasis growth of multidrug-resistance colon and breast cancer models in mice [133,134]. Moreover, this vaccine caused an up to 70% increase in the uptake of chemotherapeutic drugs [134]. Direct inhibition of FAP led to similar results in lung and colon cancer [135]. However, targeting FAP as a major fibroblast marker might give rise to adverse effects, particularly a potential deficiency in wound healing. In the mice studies conducted, no indications of impaired wound healing or autoimmune reactions were observed [133,134]. However, it remains essential for future research involving patients to ascertain whether these findings hold true within the human context.

Gene analyses revealed CXCL12 as an upregulated chemokine in breast cancers, showing a low cytotoxic T lymphocyte infiltration. Inhibition of C-X-C motif receptor 4 in fibroblasts, the receptor of CXCL12, resulted in decreased fibrosis, increased infiltration of cytotoxic T lymphocytes, and decreased immunosuppressive microenvironment in metastasized breast cancer models. Moreover, CXCR4 suppression improves the treatment response to immune checkpoint inhibitors significantly [136]. Interestingly, in other cancer entities, alternative effects could be observed. Patients with colorectal cancer, who received a complete pathologic response after immune checkpoint therapy, showed a significantly higher infiltration of CXCL12-positive fibroblasts and suppression of CC-chemokine-ligand 2 fibroblasts [137]. This underlines the theory that different cancer entities rely on different fibroblast subgroups.

Preclinical data could show a possible survival benefit for the combination of fibroblast growth factor receptor tyrosine kinase inhibitor with immune checkpoint [138]. As mentioned above, MAFs lead to a higher tissue stiffness in colorectal liver metastasis, therefore supporting tumor angiogenesis and adding to therapy resistance. Shen et al. could prove that inhibitors of the renin-angiotensin-system could decrease the stiffness of liver metastasis by inhibition of metastasis-associated fibroblasts and consequently increase the therapeutic effect of antiangiogenic therapy, like Bevacizumab [91]. Thus, targeting cancer-associated fibroblasts can not only affect tumor progression directly but can also help to overcome therapy resistance.

*Targeting Fibroblasts and Extracellular Matrix May End in Tumor Progression*

While the described therapeutic approaches hold promise, it is important to exercise caution when targeting fibroblasts and components of the extracellular matrix. This approach is a double-edged sword and requires careful consideration, particularly due to the incomplete understanding of many underlying mechanisms. Exemplary deletion of collagen type I in myofibroblasts leads to a significant reduction of collagen type I in the extracellular matrix. However, an accelerated progression of pancreatic ductal adenocarcinoma with decreased overall survival could be observed [25]. This highlights a discrepancy between fibroblast function and extracellular matrix, which is yet to be better understood.

*The Impending Task Is to Successfully Transition Experimental Therapeutic Options into Practical Clinical Applications*

As previously mentioned, a variety of innovative treatment options have emerged in recent times. Regrettably, only a handful of these have made the transition into practical clinical implementation (Table 3) [139]. Although preclinical data holds promise, clinical studies often face interruptions due to adverse events or a lack of observed patient benefits. This could be explained by the incomplete transferability of animal models to the human context. Additionally, as mentioned earlier, there is an ongoing assessment of combinations involving fibroblast inhibitors and immune-regulating medications to address therapy resistance. However, this approach raises the concern of specific activation of the immune system, potentially leading to autoimmune reactions. In forthcoming research, careful consideration must be given to this aspect. Conversely, as previously mentioned, fibroblasts constitute a diverse cellular family. This diversity results in varying treatment outcomes across distinct cancer types. For instance, in cases of advanced basal cell carcinoma, individuals treated with Vismodegib—an inhibitor of the hedgehog pathway—exhibited complete responses. Consequently, Vismodegib received official approval for therapeutic use [140]. However, Vismodegib showed no benefit for patients with pancreatic adenocarcinoma [141,142]. On the contrary, Losartan, an inhibitor of the renin-angiotensin system, showed promising results in a single-arm phase II study [143] and is currently under investigation in combination with FOLFIRINOX and Nivolumab in patients with pancreatic cancer (NCT03563248). Furthermore, in certain murine cancer models, subcutaneous injection of cancer cells is used, while others utilize orthotopic mouse models. This divergence in methodology could potentially influence the composition of fibroblasts in distinct mouse models of the same cancer type, stemming from variations in resident fibroblast populations even when employing identical cancer cells.

RO7122290, a bispecific antibody, exhibits a dual binding capacity. It engages with FAP as well as 4-1BB, offering a prospective drug for clinical exploration. Notably, 4-1BB is prevalent in immune cells such as natural killer cells. RO7122290 targets FAP-expressing cells while concurrently triggering 4-1BB-expressing immune cells, thereby amplifying the antitumoral immune response. The clinical use in patients with solid tumors, like breast, colorectal, lung, renal cell carcinoma, and others, showed a response in a phase I study [144]. Moreover, Erdafitinib, functioning as an inhibitor of fibroblast growth factor receptors, secured approval for treating individuals with advanced urothelial carcinoma. This endorsement was based on evidence from a phase II study, which demonstrated an objective tumor response rate of 40% among all participants enrolled in the study [145,146].

In summary, targeting CAFs is a promising treatment option in experimental research with the potential for clinical translation, especially in combination with already-established drugs. Supplementary clinical studies must be conducted to identify further fibroblast-related drugs suitable for clinical application. These studies should be tailored to distinct cancer entities.

**Table 3:** Exemplary drugs targeting fibroblasts, which are used or evaluated for clinical use. The list of drugs, as well as cancer entities, is not intended to be exhaustive. FAK: focal adhesion kinase, FAP: fibroblast activation protein, FGFR: fibroblast growth factor receptor, TGF-β: transforming growth factor β.

Drug Name	Target	Combination Drug	Cancer Entity	Development Phase	Reference
Defactinib	FAK	Pembrolizumab, Gemcitabine	Pancreatic adenocarcinoma	I/II	[147]
Erdafitinib	FGFR		Bladder cancer	approved	[145]
Losartan	TGF-β	FOLFIRINOX	Pancreatic adenocarcinoma	II	[143]
RO7122290	FAP	Atezolizumab	Breast, colorectal, lung, renal carcinoma	I	[144] NCT04826003
Vismodegib	Hedgehog pathway		Basal cell carcinoma	approved	[140]
			Meningioma	II	NCT02523014
		Gemcitabine	Pancreatic adenocarcinoma	discontinued	[141,142]

## 5. Conclusions

The heterogeneous cell family of fibroblasts plays a significant role in tumorigenesis and has recently been further subcategorized through single-cell analysis. The four main subtypes—eCAFs, iCAFs, myofibroblasts, and pericytes—exert influence over the tumor microenvironment and extracellular matrix by releasing cytokines, chemokines, and extracellular matrix proteins, with variations depending on the tumor entity as well as the tumor site. Fibroblasts can exhibit phenotypes displaying pro- and antitumorigenic characteristics.

The extracellular matrix is a highly intricate system composed of a variety of proteins. Although fibroblasts serve as the main source of its secretion, other cell types also play a role in shaping the overall protein composition. The extracellular matrix is the main contributor to tumor stiffness and can be altered by cancer cells as well as external conditions like hypoxia or radiotherapy. The Inhibition of key enzymes in fibroblasts leads to a reduced tumor growth progression and a decreased metastatic burden.

Given the multitude of pathways and regulatory mechanisms involved, cancer-associated fibroblasts and the extracellular matrix have emerged as promising therapeutic targets. This could put forth targeted treatment options preventing severe adverse events. For instance, inhibitors of the renin-angiotensin system, already widely utilized in the treatment of arterial hypertension, have demonstrated the ability to inhibit CAFs and collagen synthesis. Thus, this showed potential solutions to overcome therapy resistance. Nonetheless, most of the emerging treatment targets are still in the preclinical or experimental development stage. The transition from in vitro or murine studies to practical application in patients requires careful evaluation. Subsequent clinical investigations are imperative, with a focus on monitoring potential adverse events such as impaired wound healing or autoimmune reactions.

The translation of these fundamental research findings into routine clinical use would be the next challenge in advancing anticancer therapy.

## Figures and Tables

**Table 1:** Properties of the four main fibroblast groups. +: activation, -: inhibition, *: both effects were described in the literature, eCAF: extracellular matrix cancer-associated fibroblast, iCAF: inflammatory cancer-associated fibroblast, and myCAF: myofibroblast.

Effect	Myofibroblasts	Pericytes	eCAFs	iCAFs
**Cancer cells**				
Invasion	+	+	+	
Migration	+	+		
Proliferation	+			+
Stemness		+	+	
				
**Angiogenesis**	+	+		+
				
**Immune cells**				
Glucocorticoid secretion				+
Protumoral macrophages			+	+
CD4^+^ T lymphocytes				+/- *
- Effector	+			
- Regulatory	+/- *			
CD8^+^ T lymphocytes	-			
				
**Extracellular matrix**				
Collagen secretion	+			+
Fibronectin secretion		+		
Fibrosis	+			
Premetastatic niche		+	+	

**Table 2:** Different cancer entities with clinical effects of fibroblast subgroups. CAF: cancer-associated fibroblast, eCAF: extracellular matrix cancer-associated fibroblast, HAPLN1: Hyaluronan and proteoglycan link protein 1, HR: hazard ratio, iCAF: inflammatory cancer-associated fibroblast, LPP: Lipoma preferred partner, myCAF: myofibroblast, n/a: further details not available, OS: overall survival, PDPN: Podoplanin; PFS: progression-free survival, and SMA: α-smooth muscle actin, vs.: versus.

Cancer Entity	Fibroblast Subgroup	Effect (High vs. Low)	Reference
Bladder cancer	iCAF	worse survival (n/a)	[81]
	SLC14A1^+^-CAF	worse survival (n/a)	[27]
Breast cancer	myCAF	worse survival (n/a)	[47]
	PDPN^+^-CAF	worse survival (n/a)	[38]
	S100A4^+^-CAF	better survival (n/a)	[38]
			
Cholangiocarcinoma	iCAF	tumor growth	[52]
	myCAF	worse survival (5-year survival: 6% vs. 29%)	[51]
Colorectal cancer	CAF	worse survival (HR: 2.20)	[19]
	iCAF	worse survival (n/a)	[83]
Gastric cancer	eCAF	worse survival (n/a)	[24]
	HAPLN1^+^-CAF	worse survival (n/a)	[115]
	LPP^+^-CAF	worse survival (n/a)	[22]
Non-small lung cancer	myCAF	worse survival (n/a)	[40]
Ovarian cancer	Pericytes	worse survival(mean PFS: 9 vs. 29 months)	[62]
Pancreatic ductal adenocarcinoma	Periostin^+^SMA^+^-CAF	worse survival(mean OS: 26 vs. 34 months)	[18]
	myCAF	better survival (n/a)	[26]
		inducing metastasis	[50]

## Data Availability

No new data were created.
